# ESKAPE Pathogens in Bloodstream Infections: Dynamics of Antimicrobial Resistance from 2018 to 2024—A Single-Center Observational Study in Poland

**DOI:** 10.3390/jcm14196932

**Published:** 2025-09-30

**Authors:** Aneta Guzek, Dariusz Tomaszewski, Zbigniew Rybicki, Wiesław Piechota, Katarzyna Mackiewicz, Monika Konior, Anna Olczak-Pieńkowska

**Affiliations:** 1 Department of Laboratory Diagnostics, Section of Microbiology, Military Institute of Medicine—National Research Institute, 04-141 Warsaw, Poland; aguzek@wim.mil.pl (A.G.); kmackiewicz@wim.mil.pl (K.M.); 2 Department of Anesthesiology and Intensive Therapy, Military Institute of Aviation Medicine, 01-755 Warsaw, Poland; 3 Department of Anesthesiology and Intensive Therapy, Military Institute of Medicine—National Research Institute, 04-141 Warsaw, Poland; morus39@gmail.com; 4 Department of Laboratory Diagnostics, Military Institute of Medicine—National Research Institute, 04-141 Warsaw, Poland; wpiechota@wim.mil.pl; 5 Department of Epidemiology and Tropical Medicine, Military Institute of Medicine—National Research Institute, 04-141 Warsaw, Poland; mkonior@wim.mil.pl; 6 Department of Public Health, Epidemiology and Vaccinology, Military Institute of Medicine—National Research Institute, 04-141 Warsaw, Poland; aolczak-pienkowska@wim.mil.pl

**Keywords:** *Enterococcus faecium*, *Staphylococcus aureus*, *Klebsiella pneumoniae*, *Acinetobacter baumannii*, *Pseudomonas aeruginosa*, *Enterobacter*, bloodstream infections, drug resistance, multiple, bacterial, anti-bacterial agents

## Abstract

**Background/Objectives**: Modern healthcare faces a growing burden of antimicrobial resistance, prominently driven by ESKAPE pathogens. These organisms—*Enterococcus faecium*, *Staphylococcus aureus*, *Klebsiella pneumoniae*, *Acinetobacter baumannii*, *Pseudomonas aeruginosa*, and *Enterobacter* spp.—are the leading causes of healthcare-associated infections, associated with limited therapeutic options and increased morbidity. Continuous surveillance is crucial for informing empirical therapy and guiding stewardship. **Methods**: We perform a single-center, seven-year retrospective study (2018–2024) at a 1000-bed tertiary hospital in Warsaw, Poland. Bloodstream isolates of ESKAPE pathogens were identified according to the EUCAST guidelines. Data were analyzed by pathogen, ward, and year of isolation. **Results**: From 2483 positive blood cultures, 3724 ESKAPE pathogens were recovered. *S. aureus* and *K. pneumoniae* predominated, particularly in the Intensive Care Unit and Hematology ward. Resistance analysis revealed persistently high vancomycin resistance in *E. faecium*, variable but notable methicillin resistance in *S. aureus*, and frequent ESBL production in *K. pneumoniae* with an alarming rise in carbapenemase-producing strains, including dual NDM + OXA-48 co-producers. *A. baumannii* exhibited near-universal multidrug resistance. *P. aeruginosa* demonstrated lower resistance rates with preserved colistin susceptibility, while *Enterobacter* spp. remained fully carbapenem-susceptible. Linezolid retained activity against *E. faecium*, while colistin remained effective against *A. baumannii* and *P. aeruginosa*. Modern β-lactam/β-lactamase inhibitor combinations were active against *K. pneumoniae*. **Conclusions**: Our findings underscore the critical role of ESKAPE pathogens in bloodstream infections and highlight divergent resistance patterns across species. The emergence of carbapenemase-producing *K. pneumoniae* and the persistence of multidrug-resistant *A. baumannii* are of particular concern. Sustained surveillance, robust antimicrobial stewardship, and tailored infection control strategies remained essential to curb the evolving resistance threat in tertiary care settings.

## 1. Introduction

Modern healthcare is increasingly challenged by the growing threat of antimicrobial resistance, especially in hospital-acquired infections. A particularly concerning group of pathogens is collectively referred to as the ESKAPE group (*Enterococcus faecium*, *Staphylococcus aureus*, *Klebsiella pneumoniae*, *Acinetobacter baumannii*, *Pseudomonas aeruginosa*, and *Enterobacter* spp.). These organisms are known for their high potential to cause healthcare-associated infections (HAIs) and their significant resistance to available antimicrobial treatment [1].

Infections caused by ESKAPE pathogens have profound clinical and economic implications. Patients with HAIs have significantly longer hospital stays–an average of 20.3 days compared to 8.7 days for non-infected patients [2]. Specific infections, such as central line-associated bloodstream infections (CLABSI) and catheter-associated urinary tract infections (CAUTI), can prolong hospitalization by 13.4 and 8.9 days, respectively, resulting in substantial additional healthcare costs [3].

Treatment costs for patients with these infections are significantly higher. For instance, the additional cost per patient for treating CLABSI is estimated at $43,975, and for CAUTI, $31,253 [3]. Furthermore, patients in isolation due to HAIs generate daily treatment costs that are approximately €950.65 higher than those for non-infected patients [4].

These infections are also associated with increased mortality. Evidence suggests that HAIs significantly raise the risk of death, especially among patients with additional clinical risk factors such as central venous catheters, mechanical ventilation, or immunosuppression [2].

While numerous studies have documented the prevalence and resistance patterns of ESKAPE pathogens in different geographic regions, there remains a need for comprehensive, institution-specific analyses that integrate both microbiological and clinical data. Previous surveillance studies have often focused on single pathogens, specific wards, or limited patient populations, which may not capture the broader dynamics of antimicrobial resistance within a tertiary care setting. For example, a study conducted at a university hospital in Italy evaluated the epidemiology and prevalence of antimicrobial resistance in ESKAPE pathogens over five years, providing valuable insights into local resistance trends [5]. Similarly, research in Ethiopia assessed the prevalence and antimicrobial susceptibility patterns of ESKAPE pathogens among patients who developed surgical site infections, highlighting the importance of region-specific data [6].

By contrast, this study systematically examines the prevalence of all ESKAPE pathogens within a large university hospital, evaluating their antimicrobial susceptibility and resistance profiles. This dataset not only provides updated local epidemiological insights but also enables comparisons with regional and global trends, thereby informing more targeted infection control and therapeutic strategies. Understanding these patterns is crucial for optimizing antimicrobial stewardship programs and mitigating the clinical and economic impacts of hospital-acquired infections (HAIs).

The study aims to analyze the prevalence of ESKAPE pathogens in a large university hospital and evaluate their antimicrobial susceptibility and resistance. Understanding the scope of the problem is essential for developing more effective preventive and therapeutic strategies.

## 2. Materials and Methods

### 2.1. Data Collection

The study was designed as a single-center retrospective study. The project was conducted at the Microbiology Laboratory of the Military Institute of Medicine–National Research Institute in Warsaw, Poland. The Military Institute of Medicine is a 1000-bed tertiary-care university hospital and a regional trauma center.

### 2.2. Blood Cultures and Pathogen Detection

Whole blood samples were collected concurrently for microbiological diagnostics when sepsis was clinically suspected. By standard clinical procedures, one or more sets of blood cultures—each consisting of one anaerobic and one aerobic bottle—were obtained from each patient. Blood culture bottles were loaded into a BacT/ALERT Virtuo^®^ system (bioMérieux, Marcy-l’Étoile, France) and incubated at 37 °C until flagged positive or for a maximum of five days. Upon a positive signal, samples were Gram-stained and subcultured onto Columbia blood agar, chocolate agar, and MacConkey agar, while anaerobic bottles were plated on Schaedler agar. Aerobic plates were incubated at 37 °C in ambient air supplemented with 5% CO_2_, and Schaedler agar plates were incubated anaerobically at 37 °C for 48 h.

### 2.3. Identification of the Pathogens

Between 2018 and 2020, microorganisms were identified using the VITEK^®^ 2 system (bioMérieux, France). We employed GN ID Cards for fermenting and non-fermenting Gram-negative bacilli and GP ID Cards for Gram-positive bacteria. From January 2021 onward, all strains were identified by Matrix-Assisted Laser Desorption Ionization-Time Of Flight mass spectrometry (MALDI-TOF; VITEK^®^ MS, bioMérieux, France).

Non-duplicate isolates of ESKAPE pathogens were analyzed. Duplicates were defined based on unique patients’ identifiers and blood sample collection dates (within a 28-day interval). Non-ESKAPE pathogens and fungal isolates were excluded from the analysis.

### 2.4. Antimicrobial Susceptibility Testing

Antimicrobial susceptibility testing was performed using the VITEK-2 automated system (bioMérieux, France), following the manufacturer’s instructions. Colistin susceptibility was determined separately by the reference broth microdilution method, using the MICRONAUT MIC-Strip colistin (MMS) assay (Merlin Diagnostika GmbH, Bornheim, Germany).

The VITEK 2 system utilized the AST N-N331 and AST-N 332 cards for Gram-negative bacteria, and the AST-P644 and AST-643 cards for Gram-positive bacteria.

Results were interpreted according to the European Committee on Antimicrobial Susceptibility Testing (EUCAST) criteria.

Quality control testing included the following reference strains: Pseudomonas aeruginosa ATCC 27853, *Escherichia coli* ATCC 25922, *Staphylococcus aureus* ATCC 29213, *Enterococcus faecalis* ATCC 29212, *Klebsiella pneumoniae* ATCC 700603, *Klebsiella pneumoniae* ATCC BAA-2814, *Staphylococcus aureus* NCTC 124943, and *Enterococcus faecalis* ATCC 51299.

### 2.5. Detection and Resistance Mechanisms

Phenotypic detection of ESBLs was performed with double-disk synergy tests (DDST) [7].

The presence and nature of carbapenemase determinants were assessed by molecular testing of bacterial isolates using either a polymerase chain reaction (PCR)-based platform, the GeneXpert System (Cepheid, Sunnyvale, CA, USA), or a lateral flow immunochromatographic assay (RESIST-5 OOKNV, CORIS, BioConcept, Gembloux, Belgium).

Detection of methicillin resistance in *Staphylococcus aureus* (MRSA) was performed using the cefoxitin disk diffusion method. A zone diameter of less than 20 mm was interpreted as resistant [8].

Vancomycin resistance was assessed using the vancomycin E-test, performed according to the manufacturer’s instructions (bioMérieux, France). Isolates with the minimum inhibitory concentration (MIC) values of ≥32 μg/mL were confirmed as vancomycin-resistant Enterococcus (VRE).

### 2.6. Statistics and Graphics

All data preprocessing and visualizations were performed using the Anaconda Navigator environment (version 2023.07, Anaconda Inc., Austin, TX, USA) with Jupyter Notebook (version 7.4.3. Project Jupyter, Berkeley, CA, USA). Plots were generated using Python libraries, including Matplotlib (version 3.7.2), Pandas (version 2.0.3), and Seaborn (version 0.12.2).

## 3. Results

Between 1 January 2018, and 31 December 2024, ESKAPE pathogens were detected in 2483 out of all analyzed samples. These samples were collected from 1460 patients, and a total of 3724 bacterial strains were isolated. Among them, there were 508 *E. faecium* strains, 1284 *S. aureus* strains, 963 *K. pneumoniae* strains, 461 *A. baumannii* strains, 328 *P. aeruginosa* strains, and 182 *Enterococcus* spp. strains.

The Intensive Care Unit (ICU) yielded the highest number of isolates (n = 888). The most frequently isolated pathogen in the ICU was *S. aureus* (228/888, 25.7%), *K. pneumoniae* (226/888, 25.6%), *A. baumannii* (208/888, 23.4%), and *E. faecium* (59/888, 6.7%). The Hematology ward accounted for 594 isolates, predominantly *K. pneumoniae* (170/594, 28.6%) and *S. aureus* (124/594, 20.9%).

Temporal and spatial trends were observed in the frequency of pathogen isolation. The COVID ward, which was operational primarily during 2020–2021, exhibited a high prevalence of *E. faecium* (n = 47), *S. aureus* (n = 31), and *K. pneumoniae* (n = 31), with 39 out of the *E. faecium* isolates identified in 2020 alone.

In the Nephrology ward, *S. aureus* was the predominant pathogen (n = 142), while *E. faecium* was less frequent (n = 31).

In the Cardiology ward, *S. aureus* accounted for 190 isolates, significantly outnumbering other pathogens.

Throughout the study period, several temporal patterns emerged. *Acinetobacter baumannii* isolates in the ICU decreased after 2020 (34 in 2020 in comparison to 11 in 2024). *Klebsiella pneumoniae* maintained a consistently high isolation rate in both the Hematology ward and ICU. A notable increase in *E. faecium* was observed in the Hematology Department after 2020, with a peak between 2022 and 2024, accounting for 73.2% of all *E. faecium* isolates in that Department.

Less frequently detected pathogens, such as *Enterobacter* spp. remained relatively low in overall frequency, with 294 isolates (7.9% of the total), most commonly isolated in the Hematology Department (n = 67).

**Figure 1:**
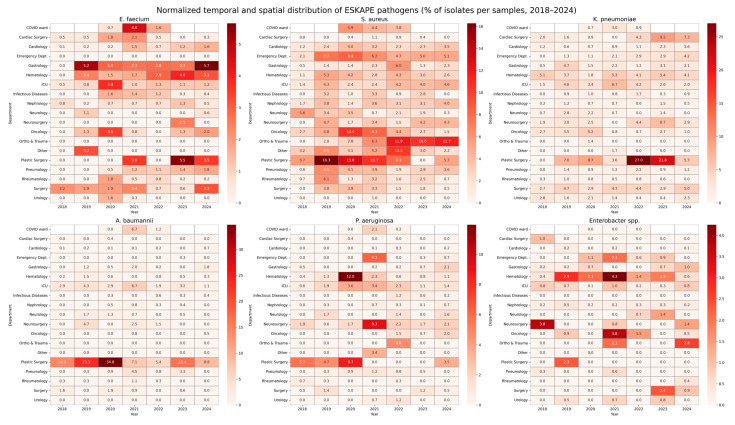
Normalized temporal and spatial distribution of ESKAPE pathogens (% of isolates per samples, 2018–2024). This figure presents heatmaps illustrating the relative prevalence of ESKAPE pathogens (*Enterococcus faecium*, *Staphylococcus aureus*, *Klebsiella pneumoniae*, *Acinetobacter baumannii*, *Pseudomonas aeruginosa*, and *Enterobacter* spp.) across hospital departments between 2018 and 2024. For each pathogen, the heatmap displays the proportion of isolates detected in a given department and year, normalized to the total number of microbiological samples collected in that department during the corresponding year. Values are expressed as percentages. Darker shades indicate a higher relative proportion of pathogen-positive samples, while lighter shades indicate lower prevalence. This visualization enables simultaneous interpretation of both the temporal dynamics and the spatial distribution of pathogens, adjusted for variations in the sampling intensity across departments.

Overall, isolates were more frequently recovered from patients located in the ICU (23.8%), with lower proportions from the Hematology (15.9%) and Cardiology (10.4%) wards. Temporal trends revealed significant fluctuation in pathogen distribution, underlying the dynamic epidemiology of antimicrobial resistance in the hospital setting. The number of isolated pathogens in each hospital department is presented in Table 1 and Figure 1.

**Figure 2:**
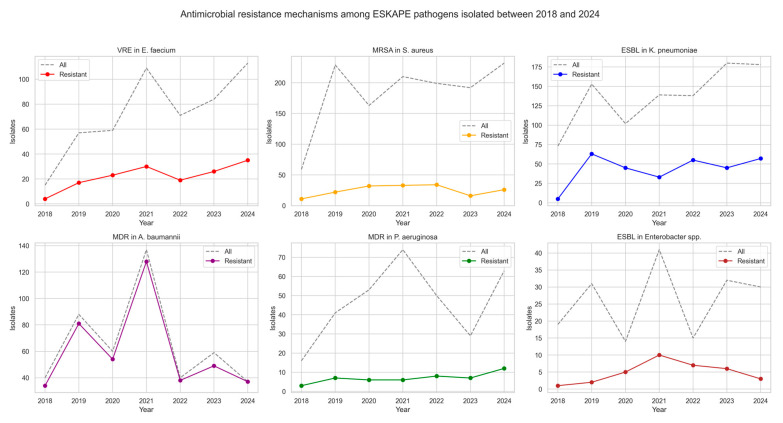
Antimicrobial resistance mechanisms of ESKAPE pathogens isolated between 2018 and 2024.

Between 2018 and 2024, ESKAPE pathogens exhibiting antimicrobial resistance were isolated, showing variable frequencies across years and species (see Table 2 and Figure 2). The proportion of vancomycin-resistant *Enterococcus faecium* (VRE) ranged between 26.7% and 39.0%, with no consistent upward or downward trend.

Methicillin-resistant *Staphylococcus aureus* (MRSA) exhibited variable prevalence, peaking in 2020 (19.6%) and reaching its lowest level in 2023 (8.3%). Among *K. pneumoniae* isolates, ESBL production remained frequent, with annual proportions ranging from 6.8% in 2018 to 44.1% in 2020. Notably, the prevalence of carbapenemase-producing *K. pneumoniae* strains increased over time. NDM-type carbapenemases were most prevalent, detected in up to 16.5% of isolates in 2021. Co-production of NDM and OXA-48 enzymes emerged in 2023 (5.6%) and persisted in 2024 (6.2%), while KPC-type carbapenemases appeared sporadically. A very high prevalence of multidrug resistance (MDR) was observed in *A. baumannii* isolates, with rates exceeding 90% in most years and reaching 100% in 2024. In contrast, *P. aeruginosa* strains were less frequent, with resistance ranging from 8.1% to 24.1% annually. Among *Enterobacter* spp. isolates, ESBL production was observed at varying frequencies, with the highest rates in 2022 (46.7%) and 2020 (35.7%), although no consistent trend was apparent over time.

**Figure 3:**
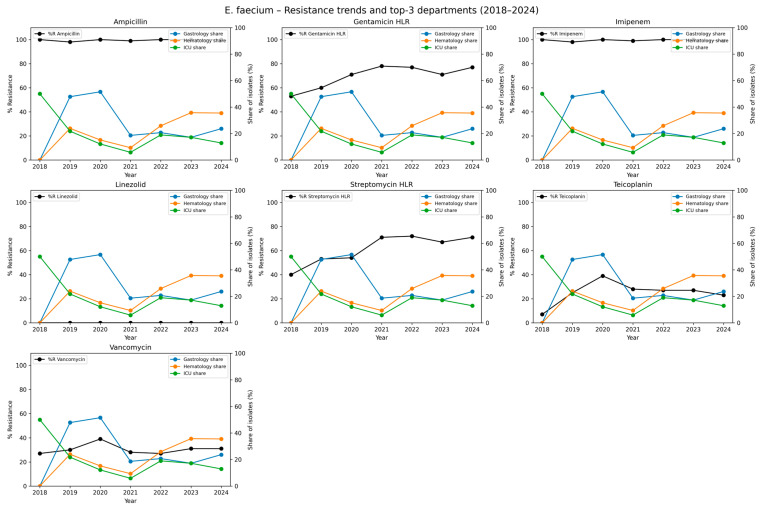
Temporal trends in antimicrobial resistance of *Enterococcus faecium* isolates and distribution across hospital departments, 2018–2024. Each subplot represents one antimicrobial agent tested against *E. faecium*. The black line shows the annual percentage of resistant isolates (%R) across all departments combined. The colored lines represent the relative contribution (share of isolates, expressed as a percentage of all *E. faecium* isolates in a given year) of the three hospital departments with the highest cumulative number of isolates across the study period. The left y-axis corresponds to the share of isolates per department (colored lines). This dual-axis approach allows simultaneous visualization of temporal resistance trends and the spatial distribution of isolates in the clinical setting. Higher %R values indicate reduced effectiveness of a given antimicrobial, while higher departmental shares indicate wards where *E. faecium* was most frequently detected.

We also analyzed the antimicrobial resistance of ESKAPE pathogens. An analysis of *E. faecium* isolates revealed persistently high resistance to ampicillin and meropenem. Resistance to ampicillin remained at 99.6% across the study period, while imipenem resistance was 99.4%, indicating near-universal resistance to β-lactam antibiotics among the isolates. High-level gentamycin resistance (HLR) increased from 53.0% in 2018 to 77.0% in 2024, resulting in an overall resistance rate of 73.0%. Similarly, high-level streptomycin resistance averaged 65.3%, increasing from 40.0% in 2018 to over 70% in the final years of our analysis. A concerning trend was observed in glycopeptide resistance. Teicoplanin resistance rose from 7.0% in 2018 to 23.0% in 2024, while vancomycin resistance increased from 27.0% to 31.0%. Notably, no resistance to linezolid was observed in any year of the study, indicating the preserved efficacy of this agent against *E. faecium* in the analyzed period. The detailed data are presented in Table 3 and graphically in Figure 3.

**Figure 4:**
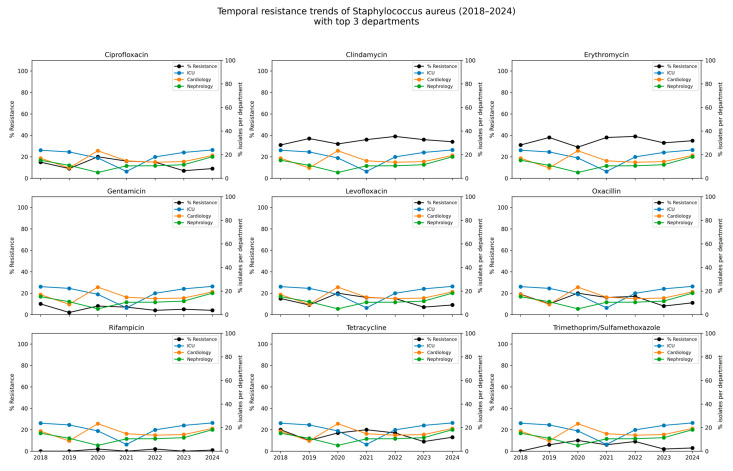
Temporal resistance trends of *Staphylococcus aureus* (2018–2024) with spatial context of isolates from the top three hospital departments. Each subplot illustrates the temporal trend in the percentage of *S. aureus* isolates resistant to a given antibiotic (black line) between 2018 and 2024. In parallel, the colored lines depict the relative contribution (%) of isolates originating from the tree hospital departments with the highest cumulative number of detections (Intensive Care Unit, Cardiology ward, Hematology ward). The left axis (black line) corresponds to the % resistance to the antibiotic, while the right axis (colored lines) shows the % share of isolates from individual departments relative to the total isolates defined in a given year.

Between 2018 and 2024, resistance to most antibiotics among *S. aureus* isolates remained low. No resistance was detected to ceftaroline, daptomycin, linezolid, teicoplanin, tigecycline, or vancomycin in any year of the study. Resistance to oxacillin (used here as a proxy for MRSA prevalence) ranged from 8.0% to 20.0%, with a mean resistance rate of 13.6%. Resistance to ciprofloxacin and levofloxacin followed similar patterns, with each reaching a peak of 20.0% in 2020 and averaging 12.3% overall. Moderate and stable resistance levels to clindamycin and erythromycin, averaging 35.4% and 35.3%, respectively, were observed over the study period. Resistance to gentamicin remained low (mean 5.1%), with the highest recorded in 2020 (8.0%). Sporadic resistance to trimethoprim/sulfamethoxazole ranged from 2.0% to 10.0%, and remained low overall (5.5%). Resistance to rifampicin was rare (0.6%) and was detected sporadically in a small number of isolates. The detailed data are presented in Table 4 and Figure 4.

**Figure 5:**
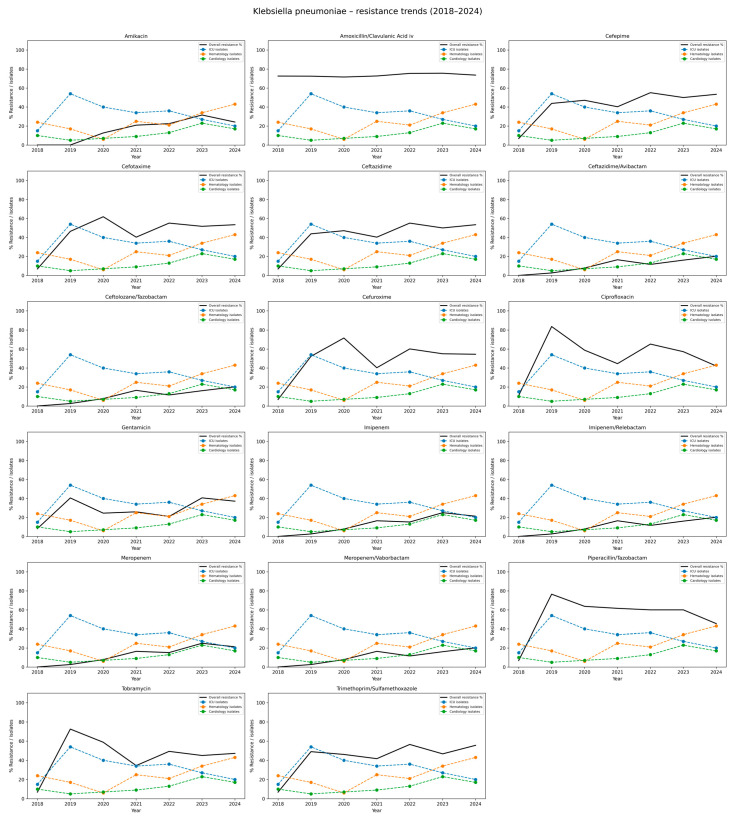
Distribution of antibiotic resistance rates of *Klebsiella pneumoniae* isolates, 2018–2024. This table summarizes both the number of isolates tested (n) and the corresponding percentage resistance (%R) for *Klebsiella pneumoniae* across the years 20182024, stratified by antibiotic. Each row corresponds to an antibiotic commonly used for testing *K. pneumoniae* susceptibility. For each year, data are presented as n (%R), where n is the number of isolates tested and %R is the percentage resistant.

Among *K. pneumoniae* isolates, high and stable resistance rates were observed for amoxicillin/clavulanate, with annual resistance ranging from 71.6% to 75.6% and an overall rate of 73.6%. Similarly, high resistance was noted for ciprofloxacin (range: 42.1–83.7%, total: 54.6%), piperacillin/tazobactam (45.5–76.5%, total: 54.2%), cefuroxime (40.3–71.6%, total: 51.2%), and trimethoprim/sulfamethoxazole (41.7–56.5%, total: 46.3%). Resistance to third- and fourth-generation cephalosporins, including cefepime, cefotaxime, and ceftazidime, increased over time and exceeded 50% in recent years (total range: 45.4–47.7%). Moderate resistance was found for gentamicin (21.0–40.6%, total: 30.8%) and tobramycin (34.5–72.5%, total: 47.5%). Resistance to amikacin remained lower, with a total rate of 18.8%, though it increased from 12.7% in 2020 to 31.6% in 2023. Resistance to carbapenems also increased over the study period, reaching a rate of over 21% in 2024 (total 14.4%). Initially, resistance to newer β-lactam/β-lactamase inhibitor combinations–such as ceftazidime/avibactam, ceftolozan/tazobactam, imipenem/relebactam, and meropenem/vaborbactam, was low; however, a gradual increase was observed from 2023 to 2024, reaching a total of 12.0%. The detailed data are presented in Table 5 and Figure 5.

**Figure 6:**
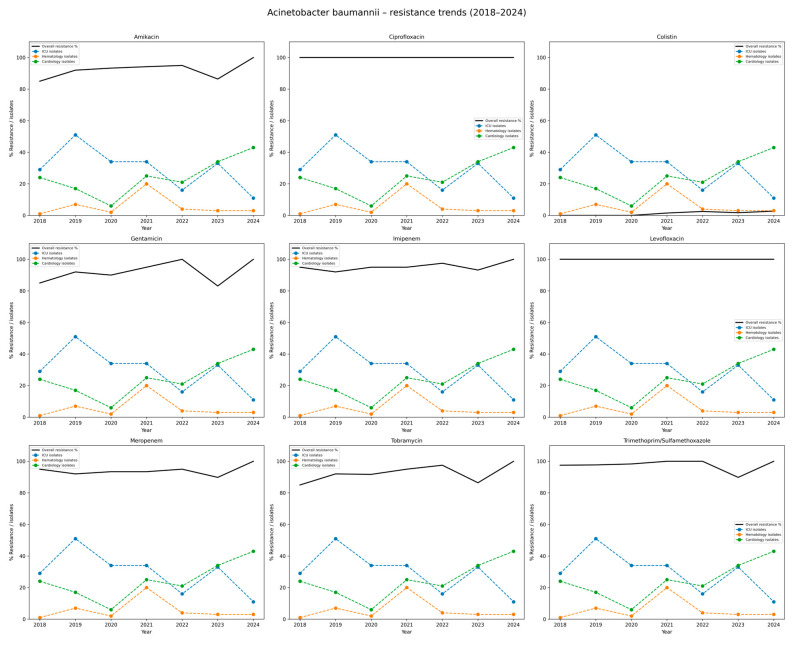
Distribution of antibiotic resistance rates of *Acinetobacter baumannii* isolates, 2018–2024. Each subplot displays the percentage resistance of *A. baumannii* to a single antibiotic. The black line depicts overall resistance rates in the hospital. The colored dashed lines correspond to the departments with the highest number of isolates (here: Intensive Care Unit, Hematology ward, Cardiology ward). The Y-axis combines % resistance and isolate counts for top departments, while the X-axis shows study years (2018–2024). The figure highlights persistently high resistance rates, particularly to carbapenems and fluoroquinolones, underlying the clinical challenge posed by multidrug-resistant *A. baumannii*.

A very high level of antimicrobial resistance was observed among *A. baumannii* isolates over the study period (Table 6 and Figure 6). Resistance to ciprofloxacin and levofloxacin remained constant at 100% in all years. Resistance to imipenem and meropenem was also consistently high, with annual resistance rates ranging from 92.0% to 100%. Similarly, resistance to gentamicin, tobramycin, and amikacin remained above 85% in most years, reaching 100% for all three agents in 2024. Resistance to trimethoprim/sulfamethoxazole was exceptionally high, ranging from 97.5% to 100%, except for a slight decrease to 89.8% in 2023. In contrast, resistance to colistin was rare, detected in only five isolates (1.1%) throughout the entire period, indicating preserved susceptibility. These findings highlight the critical threat posed by extensively drug-resistant *A. baumannii* strains, which exhibit near-universal resistance to most available antibiotics except colistin.

**Figure 7:**
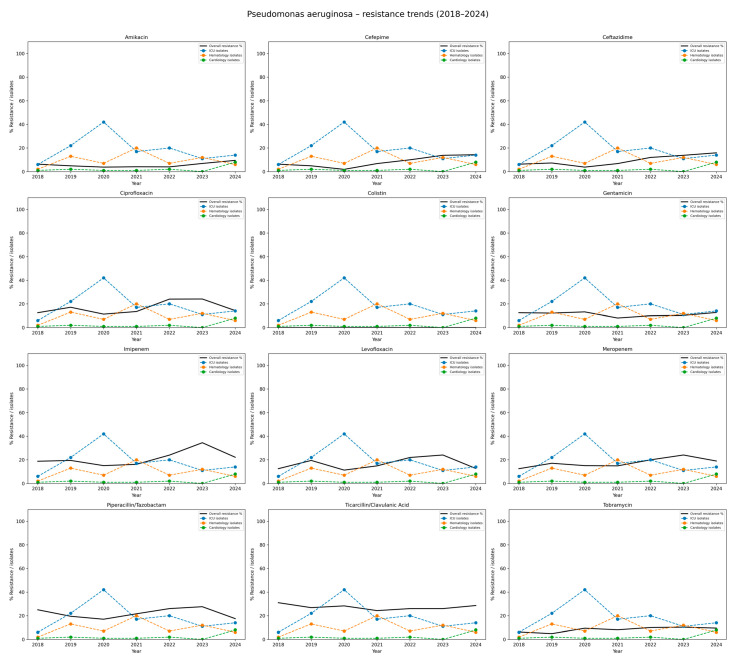
Resistance trends of *Pseudomonas aeruginosa* isolates, 2018–2024. Each subplot illustrates the resistance rate of *P. aeruginosa* to a given antibiotic. The black line indicates the overall hospital resistance percentage. The colored dashed lines represent the number of isolates reported in the three hospital departments with the highest incidence (here: Intensive Care Unit, Hematology ward, Cardiology ward). The Y-axis: percentage resistance and departmental isolate counts; the X-axis: study years (2018–2024). The figure shows considerable variability across antibiotics, with worrying levels of resistance to carbapenems and fluoroquinolones, highlighting *P. aeruginosa* as the major multidrug-resistant pathogen.

Table 7 presents the antibiotic resistance profiles of *P. aeruginosa*. The highest overall resistance was observed for ticarcillin/clavulanate, with resistance rates ranging from 26.0% to 31.0%, and a mean value of 27.3%. This pathogen also showed consistently high resistance to piperacillin/tazobactam, averaging 21.2% over the study period. Among carbapenems, resistance to imipenem increased from 18.8% in 2018 to 34.5% in 2023, with a slight decline to 22.2% in 2024. A similar trend was noted for meropenem, with resistance rising from 12.5% in 2018 to a peak of 24.1% in 2023, resulting in an average of 17.5%. Resistance levels to fluoroquinolones were variable but considerable, each averaging 16.3%. The highest resistance rate to ciprofloxacin was reported in 2022 (24.0%). Lower resistance was observed to aminoglycosides, specifically amikacin (mean 5.5%) and tobramycin (8.6%). Slightly higher but stable resistance rate to gentamicin was observed across the years, averaging 11%. Notably, no resistance to colistin was detected during the entire study period, indicating sustained susceptibility of *P. aeruginosa* to this last-resort antibiotic. Such data are graphically shown in Figure 7.

**Figure 8:**
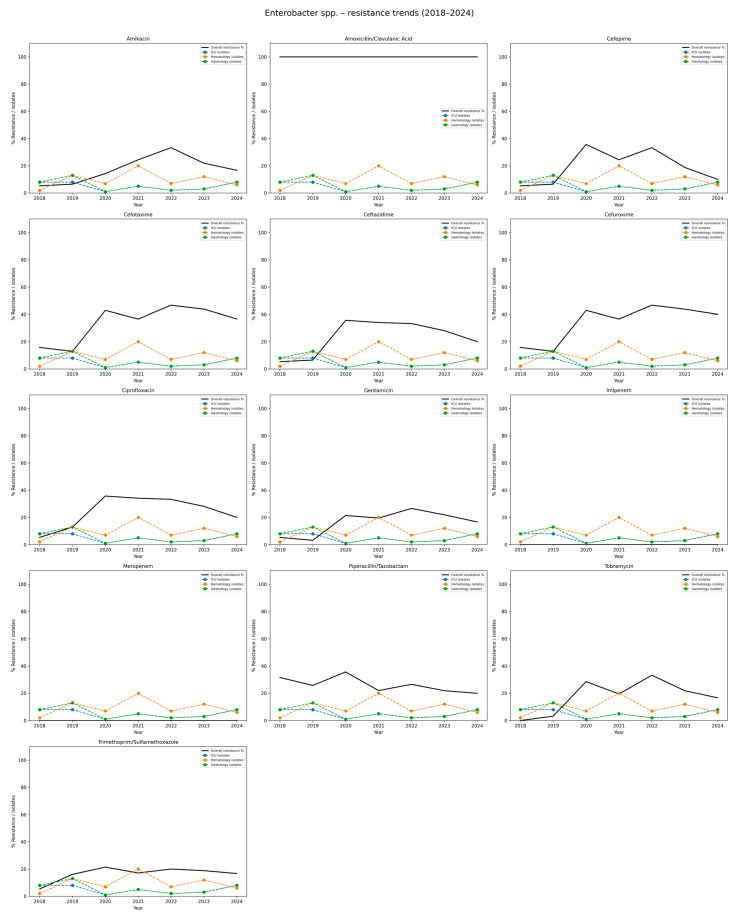
Resistance trends of *Enterobacter* spp. isolates, 2018–2024. Each subplot shows the resistance rate of *Enterobacter* spp. against a specific antibiotic. The black line depicts the overall hospital resistance percentage. The colored dashed lines represent isolate counts in the three hospital departments with the highest incidence (here: Intensive Care Unit, Hematology ward, Gastrology ward). The Y-axis: percentage resistance and isolate counts; the X-axis: study years (2018–2024). This visualization highlights the increasing resistance of *Enterobacter* spp. to multiple β-lactams and fluoroquinolones, while carbapenem resistance remained absent during the study period.

Table 8 and Figure 8 summarize the resistance profiles of *E. cloacae*. Throughout the study period, isolates showed 100% resistance to amoxicillin/clavulanate, indicating that this agent is ineffective against this species in the local setting. Among cephalosporins, resistance to cefotaxime and cefuroxime was relatively high and stable, ranging from 12.9% to 46.7%, with average resistance rates above 35%. Slightly lower but still notable resistance trends to ceftazidime (ranging from 5.3% to 33.3%), and cefepime peaking at 35.7% in 2020, were observed. Resistance to fluoroquinolones, such as ciprofloxacin, increased over time, reaching 35.7% in 2020 and remaining above 20% through 2024 (mean approximately 24.3%). Aminoglycoside resistance varied depending on the specific agent: resistance to amikacin was moderate, ranging from 5.3% to 33.3%, while resistance to gentamicin and tobramycin showed fluctuating levels, reaching peaks of 28.6% and 33.3%, respectively.

No resistance to carbapenems was detected during the entire study period, confirming the sustained effectiveness of these agents against *E. cloacae*. Piperacillin/tazobactam resistance remained relatively stable, ranging from 20.0% to 35.7% (mean ca. 26.6%). Trimethoprim/sulfamethoxazole resistance fluctuated between 5.3% and 21.4%, with no consistent trend. Overall, the data reflect a complex and variable resistance pattern of *E. cloacae*, with consistently high resistance to β-lactam/β-lactamase inhibitor combinations and moderate levels across several other antibiotic classes, excluding carbapenems.

## 4. Discussion

The analysis of ESKAPE pathogens isolated from bloodstream infections between 2018 and 2024 in our center reveals significant trends in antimicrobial resistance, aligning with global patterns and underscoring the escalating threat posed by these pathogens [9,10]. ESKAPE pathogens are responsible for 42.2 to 75.6% of bloodstream infections [11,12]. Therefore, epidemiological studies concerning ESKAPE are a key element in the prevention and treatment of hospital-acquired infections.

In our study, we analyzed the frequency of infections caused by ESKAPE bacteria across various hospital departments and evaluated the dynamics of antimicrobial resistance development over a seven-year period, from 2018 to 2024, including the COVID-19 pandemic years (2020–2022).

The study material consisted of 3724 ESKAPE isolates obtained from 2483 blood samples at the Military Institute of Medicine—National Research Institute in Warsaw, the second-largest hospital in the city. Compared to other published studies on this topic, the presented dataset can be considered representative.

The most frequently isolated pathogen was *S. aureus* (1284 isolates; 34.5%), followed by *K. pneumoniae* (963; 25.8%), *E. faecium* (508; 13.6%), *A. baumannii* (461; 12.4%), *P. aeruginosa* (328; 8.8%), and *Enterobacter* spp. (182; 4.9%). The predominance of *S. aureus* and *K. pneumoniae* in our study is consistent with the findings reported by other authors [12,13,14].

The highest number of pathogens was isolated from blood samples collected from critically ill and hematological patients, which reflects global trends and is associated with department-specific risk factors, as invasive procedures and immunosuppression.

During the COVID-19 pandemic, we observed a marked and above-average increase in infections caused by *E. faecium* (including VRE), *S. aureus*, and *K. pneumoniae*. The conclusions of other authors were similar [15,16]. We also observed that during the pandemic period, the highest number of *K. pneumoniae* ESBL and *K. pneumoniae* NDM strains were isolated. The observations from Portugal [17] and Slovakia [18] were similar. In addition, we recorded a continuous increase in multidrug-resistant (MDR) *A. baumannii* isolates, with resistance rates rising from 85% in 2018 to 100% in 2024. These trends align with recent global surveillance data [19]. This rise may be attributed to the increased number of hospitalizations, reduced availability of healthcare staff, and elevated exposure of patients to infectious agents.

We observed, for the first time in 2023, the emergence of *K. pneumoniae* strains co-producing dual carbapenemases (NDM and OXA-48), accounting for 5.6% of *K. pneumoniae* isolates, with a further increase to 6.2% in 2024. This finding aligns with reports of the global emergence and local outbreaks of NDM + OXA-48–producing *K. pneumoniae* in recent years [20,21].

Epidemiological analysis of ESKAPE bacteria is a crucial component in guiding appropriate empirical antimicrobial therapy, which is essential for treatment efficacy and reduction in mortality. An analysis conducted from 2018 to 2020 showed that approximately 32.7% of empirical antibiotic administrations were inadequate compared to subsequent microbiological findings [22].

We also analyzed the effectiveness of antimicrobial therapy in infections caused by ESKAPE pathogens. In the treatment of *E. faecium*, linezolid has been proven to be the most effective option. These observations are consistent with the conclusions of Huang et al. meta-analysis [23].

We found no resistance to ceftaroline, daptomycin, linezolid, teicoplanin, vancomycin, and tigecycline in *S. aureus*, consistent with multiple surveillance and MIC studies reporting ≥94–100% susceptibility among clinical MRSA and MSSA isolates [24].

The restricted and controlled use of last-line agents such as linezolid, vancomycin, and daptomycin likely contributes to the sustained high susceptibility of *S. aureus*. In contrast, high resistance of *E. faecium* to ampicillin is well-documented in the literature, with studies reporting approximately 80–90% of clinical isolates being resistant, making it a characteristic feature of hospital-associated strains [25].

In clinical practice, neither ampicillin nor meropenem is routinely used to treat *E. faecium* infections, as their efficacy is limited. Therefore, the presence of resistance to these agents in in vitro susceptibility testing does not impact the choice of empirical therapy. These observations highlight the importance of interpreting resistance data in the context of actual antibiotic use and stewardship practices within the hospital.

The lowest resistance rate in *K. pneumoniae*—approximately 12%—was observed for modern β-lactam/β-lactamase inhibitor combinations, including ceftazidime-avibactam, meropenem-vaborbactam, and imipenem-relebactam. These findings are consistent with data reported by Sader et al., who analyzed 35,360 multidrug-resistant *Enterobacterales* isolates collected from 75 U.S. medical centers between 2018 and 2022. In their study, ceftazidime-avibactam and meropenem-vaborbactam demonstrated susceptibility rates of 97.9%, while imipenem-relebactam showed a susceptibility rate of 93.5% [26].

Colistin remained the sole viable therapeutic option against *A. baumannii*, with resistance persisting at low levels (1.1–2.7%), in line with global surveillance reporting ~3–5% resistance [27]. *Pseudomonas aeruginosa* retained universal susceptibility to colistin throughout the study. Amikacin and cefepime showed resistance rates of 5.5% and 8.3%, respectively, which are notably lower than the typically reported rates in regional cohorts (31.1% and 44.5%) [28].

*Enterobacter* spp. demonstrated 100% susceptibility to carbapenems, which is comparable to susceptibility rates reported by Rhomberg et al. [29]. However, data from the CHINET surveillance network indicate that the percentage of carbapenem-resistant *Enterobacter* spp. reached 10%, showing an increasing trend over their 7-year study period [30].

Our study also has some limitations. The first one is its single-center design. We analyzed only isolates from blood samples, which may not fully reflect the overall spectrum of hospital-acquired infections. This study focused on the microbiological analysis of ESKAPE pathogens, including their prevalence and antimicrobial susceptibility, but did not include molecular characterization of clonal analysis. Whole-genome sequencing could provide deeper insights into resistance determinants and transmission dynamics, which will be addressed in future work.

Additionally, patient-level clinical outcomes such as mortality, length of hospital stay, and treatment success were not assessed. While these outcomes are essential for understanding the practical impact of antimicrobial resistance, they were beyond the scope of this retrospective analysis. However, despite these limitations, our data may serve as a basis for updating local empirical therapy protocols and implementing antimicrobial control strategies in a hospital setting, thereby reducing the risk of spreading multidrug-resistant bacteria. Future studies are planned to integrate molecular and clinical data, providing a more comprehensive assessment of resistance trends and their implications for patient care.

We can conclude that effective treatment of infections caused by ESKAPE pathogens relies primarily on linezolid, colistin, and modern β-lactam/β-lactamase inhibitor combinations. Among these pathogens, infections caused by *A. baumannii* pose the most significant therapeutic challenge due to the limited treatment options, which are restricted mainly to colistin, an antibiotic with significant toxic potential and poor tissue penetration.

## 5. Conclusions

Our findings confirm that multidrug-resistant ESKAPE pathogens remain a significant threat in the hospital setting. The persistence and rise in resistance mechanisms, particularly among *K. pneumoniae*, *A. baumannii*, and *E. faecium*, highlight the urgent need for continuous local and regional surveillance. The emergence of *K. pneumonia* strains co-producing dual carbapenemases (NDM + OXA-48) and the near-universal resistance of *A. baumannii* to most available antibiotics, except colistin, represent critical therapeutic challenges. These results emphasize the importance of stringent antimicrobial stewardship programs, infection control interventions, and the rational use of last-line agents such as linezolid, colistin, and modern β-lactam/ β-lactamase inhibitor combinations.

Future studies integrating molecular typing and patient-level clinical outcomes are essential to better understand resistance transmission dynamics, quantify the clinical impact of multidrug-resistant infections, and guide the development of more effective preventive and therapeutic strategies.

## Figures and Tables

**Table 1:** The number of ESKAPE pathogens isolated in different hospital departments between 2018 and 2024.

Pathogen	Year	Department																		
		ICU	Hematology	Cardiology	Gastrology	Nephrology	COVID Ward	Emergency	Pulmonology	Surgery	Plastic Surgery	Rheumatology	Oncology	Neurosurgery	Infectious Dis.	Neurology	Cardiac Surgery	Urology	Orthopedics	Others
*Enterococcus* *faecium*	2018	5	0	1	0	4	0	0	0	4	0	0	0	0	0	0	1	0	0	0
	2019	10	11	2	22	1	0	0	0	4	0	0	3	0	0	2	1	0	0	1
	2020	44	5	2	17	4	1	0	1	2	0	7	4	0	5	0	4	3	0	0
	2021	5	8	14	16	4	39	0	4	5	1	2	1	0	5	0	4	1	0	0
	2022	11	15	8	12	5	7	0	4	1	0	3	0	0	4	0	1	0	0	0
	2023	12	25	12	12	9	0	0	3	1	3	1	2	3	1	0	0	0	0	0
	2024	12	33	17	22	4	0	0	6	7	2	1	4	0	2	2	1	0	0	0
*Staphylococcus aureus*	2018	14	5	10	2	9	0	1	2	0	2	2	3	0	0	8	0	0	0	1
	2019	51	24	20	6	25	0	6	23	8	7	24	11	8	7	6	0	0	1	2
	2020	28	14	38	8	8	9	8	9	4	3	5	12	2	3	8	1	0	2	1
	2021	12	13	31	14	22	38	3	13	7	3	12	11	4	12	1	2	3	6	3
	2022	36	22	27	26	21	16	8	7	2	3	6	6	2	3	3	2	0	5	4
	2023	42	19	27	6	22	0	17	6	3	0	12	4	5	10	4	1	0	12	2
	2024	46	27	37	9	35	0	36	12	1	3	3	3	6	0	1	0	0	9	5
*Klebsiella* *pneumoniae*	2018	15	24	10	2	1	0	0	0	5	0	1	3	2	0	1	4	5	0	0
	2019	54	17	5	20	8	0	1	5	10	3	4	8	5	2	5	3	3	0	0
	2020	40	6	7	9	4	1	1	2	3	2	3	6	3	3	5	2	4	1	0
	2021	34	25	9	13	4	26	1	4	10	1	2	1	0	3	1	0	4	0	1
	2022	36	21	13	5	4	4	5	8	6	10	3	1	6	6	0	9	1	0	0
	2023	27	34	23	14	11	0	10	2	5	12	3	4	8	1	3	22	1	0	0
	2024	20	43	17	8	4	0	30	4	11	3	0	2	4	4	0	21	7	0	0
*Acinetobacter baumannii*	2018	29	1	1	0	0	0	0	0	3	5	1	0	0	0	0	0	0	0	0
	2019	51	7	2	5	0	0	0	1	0	10	1	0	8	0	3	0	0	0	0
	2020	34	2	1	3	3	0	0	2	2	8	0	0	0	1	3	1	0	0	0
	2021	34	0	1	12	5	57	0	15	2	2	4	1	3	0	1	0	0	0	0
	2022	16	4	2	1	2	5	0	3	0	2	1	0	2	2	0	0	0	0	0
	2023	33	3	0	0	3	0	1	7	1	9	0	0	0	1	1	0	0	0	0
	2024	11	3	8	7	0	0	0	0	0	5	0	1	0	2	0	0	0	0	0
*Pseudomonas* *aeruginosa*	2018	6	2	0	2	0	0	0	0	0	2	2	0	2	0	0	0	0	0	0
	2019	22	6	0	0	2	0	0	1	3	2	1	0	1	0	3	0	0	0	0
	2020	42	40	0	0	0	0	0	2	0	2	0	0	2	0	0	1	0	0	0
	2021	17	11	1	1	4	18	3	4	0	0	0	0	11	0	0	0	2	0	2
	2022	20	3	3	1	2	1	0	3	0	0	1	2	3	4	2	0	3	2	0
	2023	11	5	0	3	1	0	1	1	2	0	0	1	2	2	0	0	0	0	0
	2024	14	12	2	8	6	0	5	0	1	2	0	4	3	1	5	0	0	0	0
*Enterobacter* spp.	2018	8	2	0	1	1	0	0	1	0	0	0	0	4	0	0	2	0	0	0
	2019	8	13	2	1	3	0	0	0	0	1	0	2	0	0	0	0	1	0	0
	2020	1	7	0	4	1	0	1	0	0	0	0	0	0	0	0	0	0	0	0
	2021	5	20	2	0	1	0	1	2	0	0	0	5	1	0	0	0	2	2	0
	2022	2	7	0	0	2	0	1	0	0	0	0	2	0	0	1	0	0	0	0
	2023	3	12	0	3	2	0	3	0	4	0	0	0	0	0	3	0	2	0	0
	2024	8	6	1	4	2	0	0	0	2	0	2	1	2	0	0	0	0	2	0

The table presents the annual distribution of bloodstream isolates of ESKAPE pathogens across hospital departments in our institution from 2018 to 2024. Values represent the absolute number of isolates recovered from each department per year. Department abbreviations: ICU—Intensive Care Unit; COVID ward—dedicated COVID-19 ward; Emergency Dept.—Emergency Department; Infectious Dis.—Infectious Diseases. Other departments are written in full for clarity.

**Table 2:** Antimicrobial resistance mechanisms among ESKAPE pathogens isolated between 2018 and 2024.

Pathogen		2018	2019	2020	2021	2022	2023	2024
*Enterococcus faecium*	all	15	57	59	109	71	84	113
	VRE	4 (26.7%)	17 (29.8%)	23 (39.0%)	30 (27.5%)	19 (26.8%)	26 (31.0%)	35 (31.0%)
*Staphylococcus aureus*	all	59	229	163	210	199	192	232
	MRSA	11 (18.6%)	22 (9.6%)	32 (19.6%)	33 (15.7)	34 (17.1%)	16 (8.3%)	26 (11.2%)
*Klebsiella pneumoniae*	all	73	153	102	139	138	180	178
	ESBL	5 (6.8%)	63 (41.2%)	45 (44.1%)	33 (23.7%)	55 (39.9%)	45 (25.0%)	57 (32.0%)
	NDM	-	4 (2.6%)	8 (7.8%)	23 (16.5%)	16 (11.6%)	29 (16.1%)	25 (14.0%)
	OXA-48	-	-	-	-	4 (2.9%)	2 (1.1%)	-
	NDM + OXA48	-	-	-	-	-	10 (5.6%)	11 (6.2%)
	KPC	-	-	-	-	1 (0.7%)	4 (2.2%)	2 (1.1%)
*Acinetobacter baumannii*	all	40	88	60	137	40	59	37
	MDR	34 (85.0%)	81 (92.0%)	54 (90.0%)	128 (93.4%)	38 (95.0%)	49 (83.1%)	37 (100%)
*Pseudomonas aeruginosa*	all	16	41	53	74	50	29	63
	MDR	3 (18.8%)	7 (17.1%)	6 (11.3%)	6 (8.1%)	8 (16.0%)	7 (24.1%)	12 (19.0%)
*Enterobacter* spp.	all	19	31	14	41	15	32	30
	ESBL	1 (5.3%)	2 (6.5%)	5 (35.7%)	10 (24.4%)	7 (46.7%)	6 (18.8%)	3 (10.0%)

The table presents the annual number of bloodstream isolates of ESKAPE pathogens (all) together with the proportion of isolates exhibiting selected antimicrobial resistance mechanisms (% of all isolates per year). VRE—vancomycin-resistant *Enterococcus faecium*; MRSA—methicillin-resistant *Staphylococcus aureus*; ESBL—extended-spectrum β-lactamase–producing *Klebsiella pneumoniae* or *Enterobacter* spp.; NDM—New Delhi metallo-β-lactamase; OXA-48—OXA-48 carbapenemase; KPC—*Klebsiella pneumoniae* carbapenemase; MDR—multidrug-resistant (resistant to ≥3 antimicrobial classes).

**Table 3:** Annual distribution of antimicrobial-resistant *Enterococcus faecium* isolates, 2018–2024.

	2018	2019	2020	2021	2022	2023	2024	Total
	n (%R)							
ampicillin	15 (100.0)	56 (98.0)	59 (100.0)	108 (99.0)	71 (100.0)	84 (100.0)	113 (100.0)	506 (99.6)
gentamycin HLR	8 (53.0)	34 (60.0)	42 (71.0)	85 (78.0)	55 (77.0)	60 (71.0)	87 (77.0)	371 (73.0)
imipenem	15 (100.0)	56 (98.0)	59 (100.0)	108 (99.0)	71 (100.0)	84 (100.0)	112 (99.0)	505 (99.4)
linezolid	0 (0.0)	0 (0.0)	0 (0.0)	0 (0.0)	0 (0.0)	0 (0.0)	0 (0.0)	0 (0.0)
streptomycin HL	6 (40.0)	30 (53.0)	32 (54.0)	77 (71.0)	51 (72.0)	56 (67.0)	80 (71.0)	332 (65.3)
teicoplanin	1 (7.0)	14 (25.0)	23 (39.0)	30 (28.0)	19 (27.0)	23 (27.0)	26 (23.0)	136 (28.8)
vancomycin	4 (27.0)	17 (30.0)	23 (39.0)	30 (28.0)	19 (27.0)	26 (31.0)	35 (31.0)	154 (30.3)

Data are presented as absolute numbers of resistant isolates (n) and the percentage of resistant isolates relative to the total number of *E. faecium* isolates per year (%R). Abbreviations: HLR—High-Level Resistance; HL—High Level. Percentages are rounded to one decimal place.

**Table 4:** Annual distribution of antimicrobial-resistant *Staphylococcus aureus* isolates, 2018–2024.

	2018	2019	2020	2021	2022	2023	2024	Total
	n (%R)							
ceftaroline	0 (0.0)	0 (0.0)	0 (0.0)	0 (0.0)	0 (0.0)	0 (0.0)	0 (0.0)	0 (0.0)
ciprofloxacin	9 (15.0)	20 (9.0)	32 (20.0)	33 (16.0)	30 (15.0)	13 (7.0)	21 (9.0)	158 (12.3)
clindamycin	18 (31.0)	85 (37.0)	52 (32.0)	75 (36.0)	78 (39.0)	69 (36.0)	78 (34.0)	455 (35.4)
cloxacillin	11 (19.0)	22 (10.0)	32 (20.0)	33 (16.0)	34 (17.0)	16 (8.0)	26 (11.0)	174 (13.6)
daptomycin	0 (0.0)	0 (0.0)	0 (0.0)	0 (0.0)	0 (0.0)	0 (0.0)	0 (0.0)	0 (0.0)
erythromycin	18 (31.0)	86 (38.0)	48 (29.0)	79 (38.0)	78 (39.0)	63 (33.0)	81 (35.0)	453 (35.3)
gentamicin	6 (10.0)	5 (2.0)	13 (8.0)	15 (7.0)	8 (4.0)	9 (5.0)	9 (4.0)	65 (5.1)
levofloxacin	9 (15.0)	20 (9.0)	32 (20.0)	33 (16.0)	30 (15.0)	13 (7.0)	21 (9.0)	158 (12.3)
linezolid	0 (0.0)	0 (0.0)	0 (0.0)	0 (0.0)	0 (0.0)	0 (0.0)	0 (0.0)	0 (0.0)
oxacillin	11 (19.0)	22 (10.0)	32 (20.0)	33 (16.0)	34 (17.0)	16 (8.0)	26 (11.0)	174 (13.6)
rifampicin	0 (0.0)	0 (0.0)	3 (2.0)	0 (0.0)	3 (2.0)	0 (0.0)	2 (1.0)	8 (0.62)
teicoplanin	0 (0.0)	0 (0.0)	0 (0.0)	0 (0.0)	0 (0.0)	0 (0.0)	0 (0.0)	0 (0.0)
tigecycline	0 (0.0)	0 (0.0)	0 (0.0)	0 (0.0)	0 (0.0)	0 (0.0)	0 (0.0)	0 (0.0)
trimethoprim/sulfamethoxazole	0 (0.0)	13 (6.0)	17 (10.0)	13 (6.0)	18 (9.0)	4 (2.0)	6 (3.0)	71 (5.5)
vancomycin	0 (0.0)	0 (0.0)	0 (0.0)	0 (0.0)	0 (0.0)	0 (0.0)	0 (0.0)	0 (0.0)

Data are presented as absolute numbers of resistant isolates (n) and the percentage of resistant isolates relative to the total number of *S. aureus* isolates per year (%R). Antibiotic names are given in full for clarity. Percentages are rounded to one decimal place.

**Table 5:** Annual distribution of antimicrobial-resistant *Klebsiella pneumoniae* isolates, 2018–2024.

	2018	2019	2020	2021	2022	2023	2024	Total
	n (%R)							
amikacin	0 (0.0)	0 (0.0)	13 (12.7)	29 (20.9)	31 (22.5)	65 (31.6)	43 (24.2)	181 (18.8)
amoxicillin/clavulanate	53 (72.6)	111 (72.5)	73 (71.6)	101 (72.7)	104 (75.4)	136 (75.6)	131 (73.6)	709 (73.6)
cefepime	5 (6.8)	67 (43.8)	48 (47.1)	56 (40.3)	76 (55.1)	90 (50.0)	95 (53.4)	437 (45.4)
cefotaxime	5 (6.8)	71 (46.4)	63 (61.8)	56 (40.3)	76 (55.1)	93 (51.7)	95 (53.4)	459 (47.7)
ceftazidime	5 (6.8)	67 (43.8)	48 (47.1)	56 (40.3)	76 (55.1)	90 (50.0)	95 (53.4)	437 (45.4)
ceftazidime/avibactam	0 (0.0)	4 (2.6)	8 (7.8)	23 (16.5)	16 (11.6)	29 (16.1)	36 (20.2)	116 (12.0)
ceftolozane/tazobactam	0 (0.0)	4 (2.6)	8 (7.8)	23 (16.5)	16 (11.6)	29 (16.1)	36 (20.2)	116 (12.0)
cefuroxime	5 (6.8)	80 (52.3)	73 (71.6)	56 (40.3)	83 (60.1)	99 (55.0)	97 (54.5)	493 (51.2)
ciprofloxacin	8 (11.0)	128 (83.7)	60 (58.8)	62 (44.6)	90 (65.2)	103 (57.2)	75 (42.1)	526 (54.6)
gentamicin	6 (8.2)	62 (40.5)	25 (24.5)	36 (25.9)	29 (21.0)	73 (40.6)	66 (37.1)	297 (30.8)
imipenem	0 (0.0)	4 (2.6)	8 (7.8)	23 (16.5)	21 (15.2)	45 (25.0)	38 (21.3)	139 (14.4)
imipenem/relebactam	0 (0.0)	4 (2.6)	8 (7.8)	23 (16.5)	16 (11.6)	29 (16.1)	36 (20.2)	116 (12.0)

Data are presented as absolute numbers of resistant isolates (n) and the percentage of resistant isolates relative to the total number of *K. pneumoniae* isolates per year (%R). Antibiotic names are given in full for clarity. Percentages are rounded to one decimal place.

**Table 6:** Annual distribution of antimicrobial-resistant *Acinetobacter baumannii* isolates, 2018–2024.

	2018	2019	2020	2021	2022	2023	2024	Total
	n (%R)							
amikacin	34 (85.0)	81 (92.0)	56 (93.3)	129 (94.2)	38 (95.0)	51 (86.4)	37 (100.0)	426 (82.4)
ciprofloxacin	40 (100.0)	88 (100.0)	60 (100.0)	137 (100.0)	40 (100.0)	59 (100.0)	37 (100.0)	461 (100.0)
colistin	0 (0)	0 (0)	0 (0)	2 (1.5)	1 (2.5)	1 (1.7)	1 (2.7)	5 (1.1)
gentamicin	34 (85.0)	81 (92.0)	54 (90.0)	130 (95.0)	40 (100.0)	49 (83.1)	37 (100.0)	425 (92.2)
imipenem	38 (95.0)	81 (92.0)	57 (95.0)	130 (95.0)	39 (97.5)	55 (93.2)	37 (100.0)	437 (94.8)
levofloxacin	40 (100.0)	88 (100.0)	60 (100.0)	137 (100.0)	40 (100.0)	59 (100.0)	37 (100.0)	461 (100.0)
meropenem	38 (95.0)	81 (92.0)	56 (93.3)	128 (93.4)	38 (95.0)	53 (89.8)	37 (100.0)	431 (93.5)
tobramycin	34 (85.0)	81 (92.0)	55 (91.7)	130 (95.0)	39 (97.5)	51 (86.4)	37 (100.0)	427 (92.6)
trimethoprim/sulfamethoxazole	39 (97.5)	86 (97.7)	59 (98.3)	137 (100.0)	40 (100.0)	53 (89.8)	37 (100.0)	451 (97.8)

Data are presented as absolute numbers of resistant isolates (n) and the percentage of resistant isolates relative to the total number of *A. baumannii* isolates per year (%R). Antibiotic names are given in full for clarity. Percentages are rounded to one decimal place.

**Table 7:** Annual distribution of antimicrobial-resistant *Pseudomonas aeruginosa* isolates, 2018–2024.

	2018	2019	2020	2021	2022	2023	2024	Total
	n (%R)							
amikacin	1 (6.3)	2 (4.9)	2 (3.8)	3 (4.1)	2 (4.0)	2 (6.9)	6 (9.5)	18 (5.5)
cefepime	1 (6.3)	2 (4.9)	1 (1.9)	5 (6.8)	5 (10.0)	4 (13.8)	9 (14.3)	27 (8.3)
ceftazidime	1 (6.3)	3 (7.3)	2 (3.8)	5 (6.8)	6 (12.0)	4 (13.8)	10 (15.9)	31 (9.5)
ciprofloxacin	2 (12.5)	7 (17.1)	6 (11.3)	10 (13.5)	12 (24.0)	7 (24.1)	9 (14.3)	53 (16.3)
colistin	0 (0)	0 (0)	0 (0)	0 (0)	0 (0)	0 (0)	0 (0)	0 (0)
gentamicin	2 (12.5)	5 (12.2)	7 (13.2)	6 (8.1)	5 (10.0)	3 (10.3)	8 (12.7)	36 (11.0)
imipenem	3 (18.8)	8 (19.5)	8 (15.1)	12 (16.2)	12 (24.0)	10 (34.5)	14 (22.2)	67 (20.6)
levofloxacin	2 (12.5)	8 (19.5)	6 (11.3)	11 (14.9)	11 (22.0)	7 (24.1)	8 (12.7)	53 (16.3)
meropenem	2 (12.5)	7 (17.1)	8 (15.1)	11 (14.9)	10 (20.0)	7 (24.1)	12 (19.0)	57 (17.5)
piperacillin/tazobactam	4 (25.0)	8 (19.5)	9 (17.0)	16 (21.6)	13 (26.0)	8 (27.6)	11 (17.5)	69 (21.2)
ticarcillin/clavulanic acid	5 (31.0)	11 (26.8)	15 (28.3)	18 (24.3)	13 (26.0)	9 (31.0)	18 (28.6)	89 (27.3)
tobramycin	1 (6.3)	2 (4.9)	5 (9.4)	6 (8.1)	5 (10.0)	3 (10.3)	6 (9.5)	28 (8.6)

Data are presented as absolute numbers of resistant isolates (n) and the percentage of resistant isolates relative to the total number of *P. aeruginosa* isolates per year (%R). Antibiotic names are given in full for clarity. Percentages are rounded to one decimal place.

**Table 8:** Annual distribution of antimicrobial-resistant *Enterobacter* spp. isolates, 2018–2024.

	2018	2019	2020	2021	2022	2023	2024	Total
	n (%R)							
amikacin	1 (5.3)	2 (6.5)	2 (14.3)	10 (924.4)	5 (33.3)	7 (21.9)	5 (16.7)	32 (17.6)
amoxicillin/clavulanic acid	19 (100.0)	31 (100.0)	14 (100.0)	41 (100.0)	15 (100.0)	32 (100.0)	30 (100.0)	182 (100.0)
cefepime	1 (5.3)	2 (6.5)	5 (35.7)	10 (24.4)	5 (33.3)	6 (18.8)	3 (10.0)	32 (17.6)
cefotaxime	3 (15.8)	4 (12.9)	6 (42.9)	15 (36.6)	7 (46.7)	14 (43.8)	11 (36.7)	60 (33.0)
ceftazidime	1 (5.3)	2 (6.5)	5 (35.7)	14 (34.1)	5 (33.3)	9 (28.1)	6 (20.0)	42 (23.1)
cefuroxime	3 (15.8)	4 (12.9)	6 (42.9)	15 (36.6)	7 (46.7)	14 (43.8)	12 (40.0)	61 (33.5)
ciprofloxacin	1 (5.3)	4 (12.9)	5 (35.7)	14 (34.1)	5 (33.3)	9 (28.1)	6 (20.0)	44 (24.2)
gentamicin	1 (5.3)	1 (3.2)	3 (21.4)	8 (19.5)	4 (26.6)	7 (21.9)	5 (16.7)	29 (15.9)
imipenem	0 (0)	0 (0)	0 (0)	0 (0)	0 (0)	0 (0)	0 (0)	0 (0)
meropenem	0 (0)	0 (0)	0 (0)	0 (0)	0 (0)	0 (0)	0 (0)	0 (0)
piperacillin/tazobactam	6 (31.6)	8 (25.8)	5 (35.7)	9 (22.0)	4 (26.6)	7 (21.9)	6 (20.0)	45 (24.7)
tobramycin	0 (0)	1 (3.2)	4 (28.6)	8 (19.5)	5 (33.3)	7 (21.9)	5 (16.7)	30 (16.5)
trimethoprim/sulfamethoxazole	1 (5.3)	5 (16.1)	3 (21.4)	7 (17.1)	3 (20.0)	6 (18.8)	5 (16.7)	30 (16.5)

Data are presented as absolute numbers of resistant isolates (n) and the percentage of resistant isolates relative to the total number of *Enterobacter* spp. isolates per year (%R). Antibiotic names are given in full for clarity. Percentages are rounded to one decimal place.

## Data Availability

The data from this study are available on request from the corresponding author.

## Abbreviations

The following abbreviations are used in this manuscript:

CAUTI	Catheter-associated Urinary Tract Infections
COVID	Coronavirus Disease
CLABSI	Catheter-associated Bloodstream Infections
DDST	Double Disk Synergy Test
ESBL	Extended Spectrum β-lactamases
ESKAPE	*Enterococcus faecium*, *Staphylococcus aureus*, *Klebsiella pneumoniae*, *Acinetobacter baumannii*, *Pseudomonas aeruginosa*, and *Enterobacter* spp.
EUCAST	European Committee on Antimicrobial Susceptibility Testing
HAI	Hospital Acquired Infections
HLR	High-Level Resistance
ICU	Intensive Care Unit
KPC	*Klebsiella pneumoniae* carbapenemase
MDR	Multidrug Resistant
MIC	Minimal Inhibitory Concentration
MRSA	Methicillin-resistant *Staphylococcus aureus*
MSSA	Methicillin-susceptible *Staphylococcus aureus*
NDM	New Delhi metallo-β-lactamase
OXA	Oxacillinase
PCR	Polymerase Chain Reaction
VRE	Vancomycin-resistant *Enterococcus*
